# The Influence of a Multimodal Health Program with Diet, Art, and Biofield Therapy on the Quality of Life of People in Japan

**DOI:** 10.1089/acm.2018.0291

**Published:** 2019-03-09

**Authors:** Kiyoshi Suzuki, Tomoaki Kimura, Seiya Uchida, Hiroshi Katamura, Hideaki Tanaka

**Affiliations:** ^1^ Tokyo Ryo-in MOA Takanawa Clinic, Tokyo, Japan.; ^2^ Research Department, MOA Health Science Foundation, Tokyo, Japan.

**Keywords:** health program, integrative approach, quality of life, diet, art therapy, biofield therapy

## Abstract

***Objective:*** To investigate whether the frequency of the practice of each of diet, art, and biofield therapy influences improvement in quality of life (QOL), and to examine whether the simultaneous practice of all three components increasingly improves QOL in a real-world setting.

***Design:*** Pre–post-test design using convenience sampling methods.

***Setting:*** Home setting.

***Subjects:*** A total of 4681 individuals aged 16 years or older who answered the questionnaire appropriately.

***Intervention:*** Participants agreed to practice the three components daily and self-evaluated the frequency of their weekly practice for three consecutive months. At the beginning and end of the study, they completed the MOA quality-of-life questionnaire (10-item MOA quality-of-life questionnaire [MQL-10]).

***Outcome measures:*** Factors associated with the increase in MQL-10 scores for each component, and the relationship between the simultaneous practice of multiple components and the changes in MQL-10 scores were analyzed.

***Results:*** Frequent practice of the diet and/or art components was associated with an increase in the term-end MQL-10 score (*p* < 0.001); however, receiving biofield therapy frequently was not. Participants' age, gender, and qualification as a practitioner of biofield therapy had no relationship with changes in scores, but the reasons for participation had a significant influence on changes in scores (*p* < 0.001). Participants who initially did not practice any components frequently but who subsequently increased the number of components and frequency of each practice had a higher likelihood of exhibiting an increase in the term-end score (*p* < 0.01). Participants who initially practiced all three components frequently but later decreased the number of components practiced frequently had a lower chance of increase and a higher risk of decrease in scores (*p* < 0.01).

***Conclusions:*** The data suggest that the frequent practice of the diet and art components is associated with improvement in QOL. Simultaneous practice of diet, art, and biofield therapy is more likely to improve QOL. (ClinicalTrials.gov NCT01927250)

## Introduction

Combinations of health programs that include lifestyle interventions and psychological approaches can improve physical symptoms and quality of life (QOL) in primary health care^1,2^ and among individuals with different types of heart disease,^3,4^ diabetes,^5^ cancer,^6^ fibromyalgia,^7,8^ and depression/anxiety.^9^ In reality, people often simultaneously employ several types of health programs for health promotion and/or symptom relief.

Okada Health and Wellness Program (OHWP)^10^ is a multicomponent health program that comprises the three components of diet, art, and biofield therapy (Okada Purifying Therapy [OPT]).^10,11^ Developed in Japan, the concept of OHWP is based on the philosophy of Mokichi Okada (1882–1955). Since 2000, the MOA International Corporation (MOA)^10^ has provided OHWP as a method to promote physical–mental–spiritual well-being.

The Ministry of Agriculture, Forestry, and Fisheries of Japan^12^ and the American Academy of Nutrition and Dietetics^13^ recommend eating a sufficient amount of fresh fruits and vegetables,^14,15^ (whole) grains,^16^ using unsaturated fat-rich foods^17,18^ and less sodium^19^ for a healthy life. In addition to these points, the diet component of OHWP recommends eating organic products, which have been reported to be richer in nutritional content as compared with conventional agricultural produce.^20–24^ Organic products have also been described to stimulate plasma antioxidant activity,^22,24,25^ enhance the nutritional content of human breast milk,^26^ reduce atopic disease in infants,^27^ and lower the risk of lifestyle-related diseases.^28^ Van de Vijver and van Viet mentioned that switching to organic products was often accompanied by improvements in symptoms and lifestyle changes.^29^

According to The American Art Therapy Association, art therapy is an integrative mental health and human services profession that enriches the lives of individuals, families, and communities through active art-making, creative process, applied psychological theory, and human experience within a psychotherapeutic relationship.^30^ The British Association of Art Therapists introduces art therapy as a form of psychotherapy that uses art media as its primary mode of expression and communication.^31^ Art and/or music therapy has been reported to be effective for relieving symptoms and improving QOL in those with cancer,^32–38^ dementia,^39–41^ psychiatric diseases,^42,43^ chronic illnesses,^44^ and patients under mechanical ventilation.^45^ Ikei et al. described that viewing rose flowers changed the heart rate variability and promoted a relaxed feeling.^46^

Jain et al. explained biofields as “endogenously generated fields, which may play a significant role in information transfer processes that contribute to an individual's state of mental, emotional, physical, and spiritual well-being.”^47^ They further described biofield therapy as “noninvasive, practitioner-mediated therapies that explicitly work with the biofield of both the practitioner and recipient to stimulate a healing response in the recipient.”^47^ Biofield therapy has been reported to ameliorate pain in different illnesses,^48–52^ psychological symptoms, and/or anxiety,^48–50,53^ and to reduce agitation in individuals with dementia.^54^ In the context described by Jain et al.,^47^ OPT is a type of biofield therapy.

The general principles underlying OPT are based on Okada's concepts.^10,11^ Okada stated that synthetic substances or metabolic wastes in the body turn into toxins and cause many forms of illnesses.^10,11^ Practitioners consider stiff and/or warm spots (key areas) on the body to represent the accumulated toxins. The practitioner absorbs a certain energy from the universe and radiates it from his/her palm toward the key areas on the recipient's body. According to Okada, OPT invigorates the self-healing ability to remove accumulated toxins, thereby facilitating physical–mental–spiritual health.^10,11^

The present research team previously reported that the number of alpha waves detected by electroencephalograms increased^55,56^ and the heart rate variability in electrocardiograms^55,57,58^ changed in OPT sessions during which the recipients were unaware that they were undergoing the therapy. In a separate cross-sectional study, ∼70% of the participants reported improved symptoms after a single OPT session, although the improvement rates varied according to gender, location and duration of the session, and reasons for using OPT.^59,60^ Long-term practice of OPT has been described to improve menopausal symptoms,^61^ anemia, and survival rate in patients with sickle cell disease,^62^ and widespread pain in those with fibromyalgia.^63^

Practice of multiple components may be more effective in improving symptoms than a single component is.^64–70^ Further, the regular practice of OHWP reportedly helped ∼80% of patients with hypertension to become normotensive, and ∼30% either reduced or stopped taking medication.^71^

Beginning in 2000, MOA developed an accreditation system for OPT.^10^ Anyone can practice OPT as noncertified practitioners by taking a basic training course. To be certified as practitioners, individuals can take an advanced course and pass an examination approved by the corporation. More than 50,000 out of ∼80,000 practitioners have been certified in Japan as of June 2018.

The MOA recommends practicing the three components of OHWP simultaneously. However, individuals may practice each component with different frequencies, which may result in different outcomes regardless of the individual's favorable attitudes toward OHWP. Therefore, this study aimed (1) at investigating whether the frequency of practice of each OHWP component and/or individuals' demographic characteristics influence the extent of improvement in QOL, and (2) at examining whether the simultaneous practice of multiple components is more likely to improve QOL in a real-world setting. This study was conducted in accordance with the Helsinki Declaration of 1975, as revised in 2013, and was approved by the institutional review board and the research ethics committee of the MOA Health Science Foundation.

## Methods

### Okada Health and Wellness Program

The diet component of OHWP^10,11^ recommends eating organic produce,^72–74^ and it encourages (1) focusing on eating fresh and seasonal food,^12^ (2) avoiding using excessive seasoning,^12,19^ (3) eating meals that primarily consist of vegetables/grains,^12,14–16^ (4) eating with gratitude, and (5) engaging in appropriate diet and exercise.^2,12,65,70^ The art component encourages (1) developing sensitivity to beauty in natural/cultural environments, (2) enjoying fine art,^30^ (3) having flowers around in everyday settings,^46^ (4) attending to general personal appearance, and (5) enjoying refined forms of entertainment.^30^ As for OPT, the practitioner raises his/her hand forward toward the recipient with the palm directed toward the recipient. The practitioner uses his/her hands alternately during the administration of OPT. The distance between the palm and the body is usually 1–2 feet, with each session typically lasting 30–60 min.

The points of recommendation listed for each component originate from Okada's philosophy. They are key points that participants are encouraged to keep in mind in their daily life; thus, arranging for appropriate control groups for each component was deemed impractical. In addition, this study aimed at including as many participants as possible in a real-world setting, which would have been impaired by introducing artificially created control groups. Therefore, the research team chose a design to recruit the participants using convenience sampling methods without assigning control groups.

### Investigators

The first author delivered a lecture to ∼100 OPT instructors to explain the purpose of the study and to provide guidance for administering the questionnaires. This initial lecture was video recorded, which the instructors used to train other certified OPT practitioners as investigators. The training was conducted in 222 locations, and explanation was provided on how to instruct participants regarding OHWP practices and to use record sheets. The investigators were not financially reimbursed for conducting the OPT sessions or for participating in this study. Noncertified OPT practitioners were involved as participants and not as investigators.

### Participants

From February to December 2007, subjects received a single session of OPT from the investigators and they self-evaluated changes in symptoms after the intervention.^59,60^ They were recruited through informational materials and word-of-mouth in different settings. The inclusion criteria for participation were as follows: individuals who (1) agreed to practice OHWP in their daily lives, (2) were capable of recording the frequency of weekly practice of each OHWP component for three consecutive months, (3) were able to complete the questionnaires at the beginning and end of the study period, and (4) were aged 16 years or older. All individuals agreed to participate without receiving an honorarium. Certified practitioners could participate as both investigators and participants, provided that they met the inclusion criteria. There were no specific exclusion criteria for this study.

### Questionnaires

Participants completed a demographic questionnaire at the beginning of this study. During the study period, they self-evaluated the frequencies of their weekly practice of OHWP on a five-point scale. For the diet and art components, participants responded on the following scale once per week: always, usually, often, occasionally, and rarely. Participants also recorded how frequently they received OPT for 30 min or longer, from certified practitioners, each week, using the following five-point scale: every day, 5–6 times, 3–4 times, 1–2 times, and <once, scored 4, 3, 2, 1, and 0 points, respectively, to indicate frequency of practice. Occasionally, participants received OPT from noncertified practitioners, but these were not counted as participation because it was uncertain as to whether the study protocol was followed adequately.

To evaluate participants' QOL, a free QOL assessment questionnaire applicable to a large sample size was required. Therefore, the 10-item MOA quality-of-life questionnaire (MQL-10)^75^ was chosen for use, which was previously developed for this study (Table 1). The MQL-10 score ranges from 0 to 40, with higher scores indicating better QOL, and its minimal important difference is 3 points. The Cronbach's alpha coefficient of the MQL-10 was 0.872 at baseline and 0.879 at follow-up, both above cutoff point of 0.8, suggesting a good internal consistency. The correlation coefficient with WHOQOL-26 and SF-36 (mental health domain) was 0.81 and 0.64, respectively.^75^ Participants completed the MQL-10 questionnaire at the beginning and end of this study.

**Table 1.** Ten-Item MOA Quality-of-Life Questionnaire

Q01. How happy are you about yourself?
1 Very happy; 2 Happy; 3 Neutral; 4 Not very happy; 5 Unhappy
Q02. How satisfied are you in your everyday life?
1 Very much; 2 Satisfied; 3 Neutral; 4 Dissatisfied; 5 No satisfaction at all
Q03. How are your relationships with family, friends, and neighbors?
1 Very meaningful; 2 Meaningful; 3 No particular opinion; 4 Problematic; 5 Very difficult
Q04. How are the circumstances in your life, such as local environment, public welfare services, and the conditions of commuting to your workplace?
1 Very good; 2 Good; 3 No complaint; 4 Poor; 5 Very poor
Q05. How would you describe the present state of your health?
1 Very good; 2 Good; 3 No complaint; 4 Bad; 5 Very bad
Q06. How do you manage daily activities that involve light exercise (taking a walk, going up stairs, cleaning up, etc.)?
1 Easily; 2 Mostly easily; 3 Manageable; 4 With some difficulties; 5 Impossible
Q07. How often are you affected by physical or psychological disturbances in your daily life?
1 Not at all; 2 Occasionally; 3 Regularly; 4 Often; 5 Always
Q08. How energetic do you feel in daily activities?
1 Very; 2 Reasonably; 3 Neutral; 4 Not very; 5 Not at all.
Q09. How often do you feel anxious, depressed, or irritated?
1 Not at all; 2 Occasionally; 3 Regularly; 4 Often; 5 Very often
Q10. How often do you suffer from difficulties or stress?
1 Not at all; 2 Occasionally; 3 Regularly; 4 Often; 5 Very often

### Analyses

The frequency of OHWP was compared between the first week and in the following weeks to evaluate whether participants altered the frequency during the study period. Based on the frequency of practice in the first week, participants were divided into two groups of approximately equal number of individuals for comparison between “frequent practice” (F) and “infrequent practice” (IF) groups. The F group included participants who self-evaluated their practice of diet and/or art components either “always” or “usually,” and/or of OPT three times/week or more. The participants who reported otherwise were included in the IF group.

**FIG. 1.**
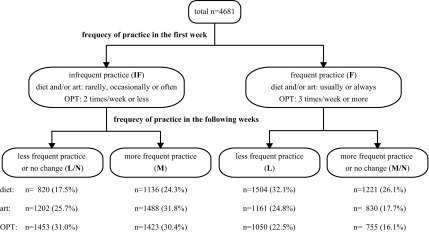
Flow chart and grouping by the practice of the Okada Health and Wellness Program: each component. OPT, Okada Purifying Therapy.

The frequency of practice in the following weeks was computed from the sum total of the weekly scores divided by the number of reports. Participants were classified into subgroups of either “less frequent practice” (L), “more frequent practice” (M), or “no change” (N) by comparing the frequencies of practice with those in the first week. Further, those who rarely practiced OHWP (score 0) in the first week were unable to report practicing less frequently during the following weeks; hence, the IF group was divided into “less frequent practice or no change” (IF-L/N) and “more frequent practice” (IF-M) subgroups. Similarly, those who always practiced OHWP in the first week (score 4) were unable to report practicing more frequently during the following weeks; therefore, the F group was divided into “less frequent practice” (F-L) and “more frequent practice or no change” (F-M/N) subgroups (Fig. 1).

**FIG. 2.**
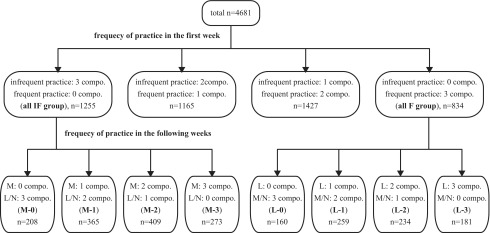
Flow chart and grouping by the practice of the Okada Health and Wellness Program: combination of three components. compo., component(s); F, frequent practice; IF, infrequent practice; L, less frequent practice; M, more frequent practice; N, no change.

Concurrently, to investigate the relationship between the frequency of the practice of multiple OHWP components and the changes in the MQL-10 scores, individuals who did not practice any components frequently in the first week (all IF group) were classified into the following four subgroups according to the number of components they practiced more frequently in the following weeks: M-0, M-1, M-2, and M-3 subgroups. Those who practiced three components frequently at the initial stage (all F group) were also classified into the following four subgroups based on the number of components they practiced less frequently in the following weeks: L-0, L-1, L-2, and L-3 subgroups (Fig. 2).

As the minimal important difference of the MQL-10 score is 3 points,^75^ the term-end score was considered to increase or decrease when the difference from the initial score was 3 points or more; otherwise, the value was evaluated as “no change.” Data were analyzed from individuals whose initial MQL-10 scores were between 3 and 37, and who filled out the frequency of OHWP practice in the first week as well as seven times or more in the following 11 weeks (two thirds or more in the 12 weeks).

The authors used the Japanese SPSS version 25 (IBM SPSS Statistics, Tokyo, Japan) for data analyses. A logistic regression analysis was conducted to examine the factors associated with the increase in MQL-10 score. The category of “decrease in the score” was used as the reference, and the initial score was analyzed as a covariate. A *p*-value of <0.05 was considered statistically significant. The Kruskal-Wallis test was employed to compare ordinal variables between four groups. If results indicated statistical significance (*p* < 0.05), the Mann-Whitney test was further conducted to compare two groups. For this, a *p*-value of <0.0083 was considered statistically significant according to the Bonferroni correction.

## Results

Of the 5637 participants who successfully reported the frequency of weekly OHWP practice eight times or more in the 12 weeks, 4681 (83.0%) met the inclusion criteria for the analyses. Table 2 indicates the basic characteristics of the participants included in the analyses. Figures 1 and 2 indicate the number of participants in each subgroup. The mean ± standard deviation of the initial and term-end MQL-10 scores were 26 ± 6 and 28 ± 5, respectively. Compared with initial scores, the term-end MQL-10 score increased in 1686 (36.0%) participants, no changes were observed in 2312 (49.4%), and it decreased in the remaining 683 (14.6%) individuals.

**Table 2.** Basic Characteristics of the Participants

Age (*n* = 4681)		
16–49	1120	(23.9%)
50–69	2100	(44.9%)
≥70	1461	(31.2%)
Female gender (*n* = 4616)	3204	(69.4%)
Reasons for participation (*n* = 4558)		
Health promotion	1980	(43.5%)
Symptom relief	1264	(27.7%)
Others	1314	(28.8%)
OPT practitioner (*n* = 4645)	3642	(78.4%)

Categorical values are shown as numbers and proportions (%).

OPT, Okada Purifying Therapy.

### Diet component

**FIG. 3.**
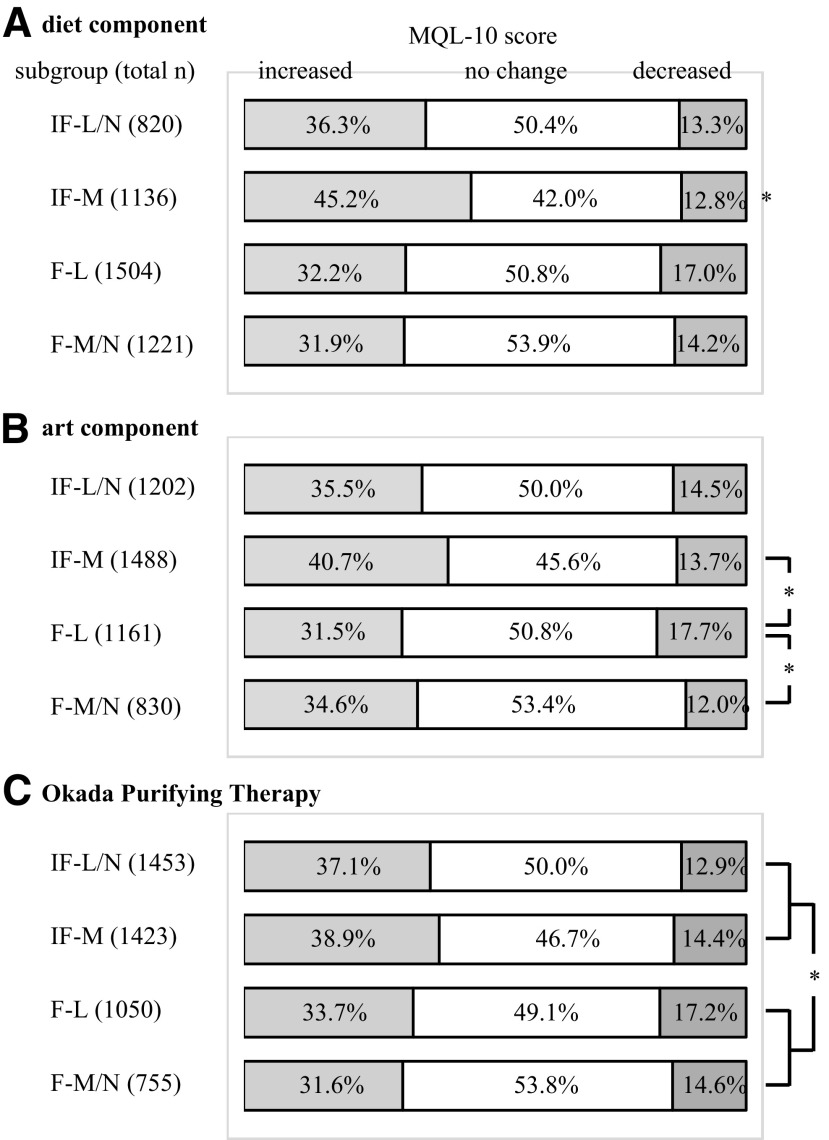
Change in the MQL-10 score between the subgroups of **(A)** diet, **(B)** art, and **(C)** OPT components. IF-L/N, those who infrequently practiced initially and less frequently practiced or no change afterward; IF-M, those who infrequently practiced initially and more frequently practiced afterward; F-L, those who frequently practiced initially and less frequently practiced afterward; F-M/N, those who frequently practiced initially and more frequently practiced or no change afterward, *p < 0.0083 by the Mann-Whitney test. F, frequent practice; IF, infrequent practice; L, less frequent practice; M, more frequent practice; MQL-10, 10-item MOA quality-of-life questionnaire; N, no change; OPT, Okada Purifying Therapy.

The IF-M subgroup included higher percentages of participants whose term-end MQL-10 scores increased more than the other subgroups (Fig. 3A). The logistic regression analysis indicated that participating for reasons other than symptom relief, more frequent practice in the following weeks, and a low initial MQL-10 score were significantly associated with the increase in the term-end scores (Table 3).

**Table 3.** Logistic Regression Analysis of the Factors Associated with an Increase in the Term-End MQL-10 Score in the Diet Component

	p*-Value*	*Odds (95% CI)*
Age	0.90	
Female gender	0.18	
OPT practitioner	0.18	
Reasons for participation		
Health promotion		1
Symptom relief	<0.001	0.60 (0.47–0.79)
Others	0.20	
Practice in the first week	0.064	
Practice in the following weeks		
Less frequent		1
No change	<0.001	1.72 (1.32–2.23)
More frequent	<0.001	2.26 (1.59–3.20)
Initial MQL-10 score	<0.001^a^	

Reference category: decrease of the term-end MQL-10 scores.

^a^ Analyzed as a covariate.

CI, confidence interval; MQL-10, 10-item MOA quality-of-life questionnaire; OPT, Okada Purifying Therapy.

### Art component

The F-L subgroup included a lower percentage of participants whose scores increased and more participants whose scores decreased than the IF-M and F-M/N subgroups (Fig. 3B). The logistic regression analysis indicated that participating for reasons other than symptom relief, frequent practice in the first week, more frequent practice in the following weeks, and a low initial MQL-10 score were independent factors that were significantly associated with the increase in the term-end score (Table 4).

**Table 4.** Logistic Regression Analysis of the Factors Associated with an Increase in the Term-End MQL-10 Score in the Art Component

	p*-Value*	*Odds (95% CI)*
Age	0.78	
Female gender	0.051	
OPT practitioner	0.15	
Reasons for participation		
Health promotion		1
Symptom relief	<0.001	0.60 (0.46–0.78)
Others	0.22	
Practice in the first week		
Infrequent		1
Frequent	<0.001	1.78 (1.39–2.30)
Practice in the following weeks		
Less frequent		1
No change	<0.001	2.06 (1.59–2.66)
More frequent	<0.001	3.03 (2.19–4.19)
Initial MQL-10 score	<0.001^a^	

Reference category: decrease of the term-end MQL-10 scores.

^a^ Analyzed as a covariate.

CI, confidence interval; MQL-10, 10-item MOA quality-of-life questionnaire; OPT, Okada Purifying Therapy.

### Okada Purifying Therapy

The IF-L/N and IF-M subgroups included higher percentages of participants whose scores increased than the F-L and F-M/N subgroups (Fig. 3C). The logistic regression analysis indicated that participating for reasons other than symptom relief and a low initial MQL-10 score were independently associated with the significant increase in the term-end score (Table 5).

**Table 5.** Logistic Regression Analysis of the Factors Associated with an Increase in the Term-End MQL-10 Score in the Okada Purifying Therapy Component

	p*-Value*	*Odds (95% CI)*
Age	0.99	
Female gender	0.11	
OPT practitioner	0.21	
Reasons for participation		
Health promotion		1
Symptom relief	<0.001	0.60 (0.46–0.78)
Others	0.11	
Practice in the first week	0.14	
Practice in the following weeks	0.72	
Initial MQL-10 score	<0.001^a^	

Reference category: decrease of the term-end MQL-10 scores.

^a^ Analyzed as a covariate.

CI, confidence interval; MQL-10, 10-item MOA quality-of-life questionnaire; OPT, Okada Purifying Therapy.

### All IF group

**FIG. 4.**
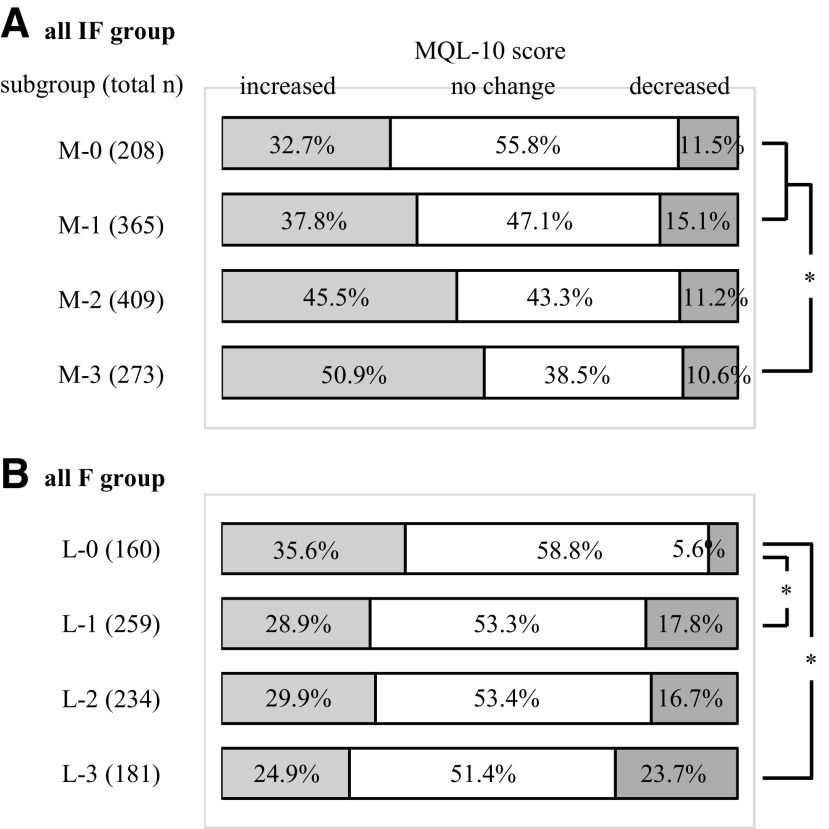
Change in the MQL-10 score between the subgroups of **(A)** all IF group and **(B)** all F group. Among participants who did not frequently practice any components initially, M-0, those who did not subsequently practice any components more frequently; M-1, those who subsequently practiced one component more frequently; M-2, those who subsequently practiced two components more frequently; and M-3, those who subsequently practiced all three components more frequently. Among participants who frequently practiced all three components initially, L-0, those who did not subsequently practice any components less frequently; L-1, those who subsequently practiced one component less frequently; L-2, those who subsequently practiced two components less frequently; and L-3, those who subsequently practiced all three components less frequently, *p < 0.0083 by the Mann-Whitney test. F, frequent practice; IF, infrequent practice; L, less frequent practice; M, more frequent practice; MQL-10, 10-item MOA quality-of-life questionnaire.

Participants who initially did not practice any components frequently but who subsequently increased the number of components and frequency of each practice had a higher likelihood of exhibiting an increase in the term-end score (Fig. 4A). The logistic regression analysis indicated that more frequent practice of all three components in the following weeks and a low initial MQL-10 score were independently associated with the increase in the term-end score (Table 6).

**Table 6.** Logistic Regression Analysis of the Factors Associated with an Increase in the Term-End MQL-10 Score in the All Infrequent Practice Group

	p*-Value*	*Odds (95% CI)*
Age	0.60	
Female gender	0.97	
OPT practitioner	0.39	
Reasons for participation	0.47	
Practice in the following weeks		
M-0		1
M-1	0.78	
M-2	0.055	
M-3	0.005	2.77 (1.36–5.65)
Initial MQL-10 score	<0.001^a^	

Reference category: decrease of the term-end MQL-10 scores.

^a^ Analyzed as a covariate.

CI, confidence interval; MQL-10, 10-item MOA quality-of-life questionnaire; OPT, Okada Purifying Therapy.

### All F group

Participants who initially practiced all three components frequently but eventually decreased the number of components practiced frequently had a lower likelihood of exhibiting an increase in and a higher risk of decrease in the term-end score (Fig. 4B). The logistic regression analysis indicated that more frequent practice or maintaining the frequency of practice of each component in the following weeks, and a low initial MQL-10 score were independently associated with the increase in the term-end score (Table 7).

**Table 7.** Logistic Regression Analysis of the Factors Associated with an Increase in the Term-End MQL-10 Score in the All Frequent Practice Group

	p*-Value*	*Odds (95% CI)*
Age	0.55	
Female gender	0.90	
OPT practitioner	0.81	
Reasons for participation	0.42	
Practice in the following weeks		
L-0		1
L-1	0.001	0.21 (0.09–0.54)
L-2	0.001	0.20 (0.08–0.51)
L-3	<0.001	0.15 (0.06–0.41)
Initial MQL-10 score	<0.001^a^	

Reference category: decrease of the term-end MQL-10 scores.

^a^ Analyzed as a covariate.

CI, confidence interval; MQL-10, 10-item MOA quality-of-life questionnaire; OPT, Okada Purifying Therapy.

## Discussion

Diet intervention or diet with exercise aims at preventing/improving lifestyle related diseases,^14,15,17–19,28,29^ which is the key to maintaining physical health. Art and/or music can be used to promote mental well-being, and it enriches the lives of individuals, families, and communities.^30,31^ Biofield therapy is often expected to contribute to the physical,^48–52,59–63^ mental,^48–50,53,59,60^ and spiritual well-being.^47^ A combination of these three health programs may be an ideal method for whole person health.

Previous studies have examined the effects of combinations of diet and exercise programs^2,65,70,76–79^; diet and mind–body practice^3^; mind–body practice and biofield therapy^66,68,80–83^; and diet, exercise, and mind–body practice^1,5,6,9^ among those with various illnesses. To the extent of the research team's knowledge, this is the first study examining the impact of a combination of diet, mind–body practice, and biofield therapy.

Frequent practice of the diet and/or art components was an independent factor associated with the increase in the term-end MQL-10 score. However, the frequency of receiving OPT had no association. Such a difference may have been caused by how each component takes shape. The diet and art components are practices requiring participants' own initiative. The OPT is a passive practice in which recipients sit/lie in front of the practitioner to receive the therapy. Some participants may have reduced the frequency of receiving OPT after their symptoms improved. Others may have received OPT more frequently with the hope of alleviating the existing symptoms. The practitioners' skillset, duration of OPT, location of administration, and practitioner–recipient relationship may also have influenced the outcome.^47,57,59,60,80^

Multimodal health programs have been reported to be more effective than a single program is for symptom relief or disease prevention.^64–70^ This study indicated that the more components individuals subsequently practiced more frequently, the higher was the likelihood of increase in the term-end score. In contrast, the likelihood of an increase in the term-end score became lower and the risk of a decrease in the score became higher as the number of components practiced frequently decreased. These findings suggest that the simultaneous practice of all three components of OHWP is more likely to improve QOL among individuals across different demographic backgrounds.

Participation for symptom relief was the negative factor associated with the increase in scores for each component. To improve symptoms and QOL, individuals may have to practice OHWP more intensively with/without help from others rather than to practice it the way they prefer in a real-world setting. The initial MQL-10 score was consistently associated with the change in the term-end score, which is considered as a regression effect.^84^ Participants' demographic differences have been reported to influence the outcomes of interventions.^3,40,47,59,60,80,85^ However, in this study, neither gender, nor age, nor the license of the OPT practitioner was associated with the changes in the participants' scores. The effectiveness of multicomponent health programs may not differ among those with various demographic characteristics.

There were several limitations in the study design. First, the survey was not experimental as it used a convenience sample without an appropriate control or comparison group. In addition, this study included a high percentage of OPT practitioners and elderly and/or female participants, both of which may have created a bias. Second, the participants may not have correctly self-evaluated the frequency of practice of each OHWP component. For example, there are no standard methods to measure the frequency of “eating with gratitude” or “enjoying fine art.” Third, the factors that were assessed in the questionnaire were not exhaustive, and other factors may have contributed to the self-reported QOL scores. Fourth, the MQL-10 questionnaire may have reflected QOL less precisely than other, more validated QOL measurement tools. Additional studies with rigorous study protocols are warranted to further investigate the effectiveness of OHWP.

## Conclusions

Among individuals with different demographic characteristics, frequent practice of diet and/or art components was associated with an increase in the term-end MQL-10 scores. However, receiving OPT frequently had no association. The chance of increasing the term-end MQL-10 score became higher as individuals practiced more components of the diet, art, and biofield therapy frequently. Participants who exhibited a decrease in the number of components practiced frequently had a lower likelihood of increase and a higher risk of decrease in scores.
